# 肺腺癌糖酵解重编程：分子机制、代谢标志物及靶向治疗策略

**DOI:** 10.3779/j.issn.1009-3419.2026.102.07

**Published:** 2026-04-20

**Authors:** Caini ZHANG, Yu FU, Huiqin FENG, Yuqing PAN, Ya LI, Chenglu HE

**Affiliations:** ^1^650032 昆明，昆明医科大学第一附属医院医学检验科; ^1^Department of Clinical Laboratory, The First Affiliated Hospital of Kunming Medical University, Kunming 650032, China; ^2^650032 昆明，云南省检验医学重点实验室; ^2^Yunnan Key Laboratory of Laboratory Medicine, Kunming 650032, China; ^3^650032 昆明，云南省医学检验临床医学研究中心; ^3^Yunnan Province Clinical Research Center for Laboratory Medicine, Kunming 650032, China; ^4^650021 昆明，云南大学附属医院检验科; ^4^Department of Laboratory Medicine, The Affiliated Hospital of Yunnan University, Kunming 650021, China

**Keywords:** 肺肿瘤, 糖酵解, 乳酸化, 代谢标志物, 代谢重编程, 肿瘤微环境, 精准诊疗, Lung neoplasms, Glycolysis, Lactylation, Metabolic biomarker, Metabolic reprogramming, Tumor microenvironment, Precision medicine

## Abstract

肺腺癌（lung adenocarcinoma, LUAD）是非小细胞肺癌中最常见且侵袭性较强的亚型，其发生发展伴随以糖酵解增强为特征的代谢重编程。糖酵解关键酶[己糖激酶2（hexokinase 2, HK2）、丙酮酸激酶M2（pyruvate kinase M2, PKM2）、乳酸脱氢酶A（lactate dehydrogenase A, LDHA）]高表达及乳酸积累不仅满足肿瘤能量与生物合成需求，还通过乳酸介导的免疫抑制及组蛋白乳酸化等表观遗传调控促进肿瘤进展与免疫逃逸。磷脂酰肌醇3-激酶/蛋白激酶B/哺乳动物雷帕霉素靶蛋白（phosphatidylinositol 3-kinase/protein kinase B/mammalian target of rapamycin, PI3K/Akt/mTOR）、缺氧诱导因子-1α（hypoxia-inducible factor-1α, HIF-1α）及MYC原癌基因（MYC proto-oncogene, c-Myc）等信号通路协同驱动糖酵解激活，重塑肿瘤免疫微环境并影响治疗响应。近年来，糖酵解相关代谢酶及影像学参数在LUAD早期诊断、预后评估和疗效监测中显示出潜在价值，多组学整合进一步推动其临床转化。糖酵解重编程不仅是LUAD的重要代谢特征，也是连接免疫抑制、治疗耐药与精准诊疗的关键环节。本文综述其分子机制、相关标志物及靶向策略，以期为早期筛查、风险分层及代谢靶向治疗提供参考。

## Abstract (en)

Lung adenocarcinoma (LUAD) is the most common and highly aggressive subtype of non-small cell lung cancer, characterized by metabolic reprogramming with enhanced glycolysis. Upregulation of key glycolytic enzymes [hexokinase 2 (HK2), pyruvate kinase M2 (PKM2), lactate dehydrogenase A (LDHA)] and lactate accumulation not only support tumor energy production and biosynthesis but also promote tumor progression and immune evasion through lactate-mediated immunosuppression and epigenetic regulation such as histone lactylation. Oncogenic signaling pathways, including phosphatidylinositol 3-kinase/protein kinase B/mammalian target of rapamycin (PI3K/Akt/mTOR), hypoxia-inducible factor-1α (HIF-1α), and MYC proto-oncogene (c-Myc), synergistically drive glycolytic activation, thereby reshaping the tumor immune microenvironment and influencing therapeutic responses. In recent years, glycolysis-related metabolic enzymes and imaging parameters have shown promising potential in the early diagnosis, prognostic evaluation, and treatment monitoring of LUAD, with multi-omics integration further facilitating their clinical translation. Collectively, glycolytic reprogramming is not only a hallmark metabolic feature of LUAD but also a critical nexus linking immunosuppression, therapeutic resistance, and precision medicine. This review summarizes the molecular mechanisms, associated biomarkers, and targeted strategies of glycolytic reprogramming, aiming to provide insights for early screening, risk stratification, and metabolism-targeted therapies in LUAD.

肺腺癌（lung adenocarcinoma, LUAD）是全球发病率最高的肺癌组织学类型，也是肺癌相关死亡的主要亚型之一^[1]^。2020年全球185个国家和地区LUAD均呈高发趋势，在东亚地区（包括中国）其年龄标化发病率位居全球前列^[2]^。过去30年，我国LUAD疾病负担持续上升，尤其在女性及从不吸烟人群中增幅显著，提示环境暴露及遗传背景等因素可能具有一定人群特异性^[3]^。在生物学层面^[4]^，LUAD除表现出复杂的驱动基因突变谱外，还呈现显著的代谢可塑性。代谢重编程，尤其是对糖酵解通路的增强依赖，被认为是肿瘤细胞适应缺氧与营养压力、维持持续增殖的重要机制之一。这种代谢适应不仅满足能量与生物合成需求，还通过调节肿瘤免疫微环境（tumor immune microenvironment, TIME）与信号网络参与疾病进展。

尽管分子靶向治疗和免疫治疗改善了部分患者的生存结局，但LUAD仍普遍存在疗效异质性与获得性耐药问题^[5]^。不同分子亚型之间的代谢依赖差异进一步影响肿瘤增殖能力、TIME特征及治疗敏感性^[6,7]^。传统影像学与组织病理学评估难以动态反映肿瘤代谢状态，限制了精准分型与个体化干预策略的优化。因此，系统阐明糖酵解等关键代谢通路的调控机制，并筛选相关代谢标志物，对于提升LUAD早期诊断、风险分层及精准治疗具有重要意义。尽管糖酵解重编程在多种实体瘤中具有共性，但LUAD在非小细胞肺癌（non-small cell lung cancer, NSCLC）中占比最高，分子分型体系最为成熟，代谢特征与治疗决策之间的关联更具明确转化场景。本文以LUAD为主要研究对象，相关机制背景在必要时参考NSCLC或其他癌种研究，但结论外推均以LUAD证据为依据。

## 1 LUAD糖酵解重编程的分层调控机制

### 1.1 代谢执行层：关键酶与代谢产物驱动

LUAD糖酵解增强的直接表型表现为代谢通量持续升高，其近端驱动来源于关键限速酶与代谢分流节点的异常激活。丙酮酸脱氢酶激酶1（pyruvate dehydrogenase kinase 1, PDK1）通过抑制丙酮酸脱氢酶复合体（pyruvate dehydrogenase complex, PDC）活性，减少丙酮酸进入线粒体氧化磷酸化通路的比例，从而促进碳流向乳酸生成方向偏移；乳酸脱氢酶A（lactate dehydrogenase A, LDHA）作为末端关键酶，维持丙酮酸还原及NAD^+^再生，保障高糖酵解通量的持续运行。研究^[8]^表明，PDK1/LDHA轴与LUAD代谢重编程及肿瘤细胞增殖、存活密切相关。

在糖酵解“入口——出口”关键节点，己糖激酶2（hexokinase 2, HK2）与丙酮酸激酶M2（pyruvate kinase M2, PKM2）在LUAD中常呈高表达或功能增强状态，可提高葡萄糖磷酸化效率并调控代谢中间产物分配，使上游代谢物积累并转入核苷酸、脂质及氨基酸等合成途径，从而满足快速增殖所需的生物合成需求^[9]^。因此，关键代谢酶的异常激活通过“代谢分流重定向——通量提升——合成底物供给”的机制，构成LUAD糖酵解重编程的细胞内在执行基础，并为代谢标志物筛选及代谢靶向干预提供直接分子依据。

### 1.2 信号调控层：核心通路的转录放大与代谢级联

在代谢执行层之上，多条信号通路通过转录与翻译层面的调控对糖酵解程序进行放大与整合。磷脂酰肌醇3-激酶/蛋白激酶B/哺乳动物雷帕霉素靶蛋白（phosphatidylinositol 3-kinase/protein kinase B/mammalian target of rapamycin, PI3K/Akt/mTOR）信号通路被认为是肿瘤代谢重编程的重要调控轴，其异常激活可通过增强葡萄糖摄取并上调多种糖酵解相关酶（如HK2、LDHA）的表达，推动肿瘤细胞代谢通量重编程^[10,11]^。在缺氧环境中，缺氧诱导因子-1α（hypoxia inducible factor-1α, HIF-1α）稳定积累，进一步诱导PDK1、LDHA等代谢基因表达，使肿瘤细胞在低氧条件下仍维持较高糖酵解活性^[12]^。此外，MYC原癌基因（MYC proto-oncogene, *c-Myc*）作为代谢重编程的关键转录调控因子，可通过转录激活LDHA等糖酵解相关基因，并通过调控剪接因子网络促进PKM2优势表达，从而增强有氧糖酵解通量并强化Warburg代谢表型^[13]^；研究^[13,14]^进一步表明，c-Myc介导的转录/转录后调控可协同上调HK2等关键代谢节点，构成糖酵解重编程的重要驱动轴。

除经典代谢调控轴外，Notch信号通路的异常激活可通过调控转录共激活因子TAZ介导糖酵解相关基因的表达，并参与肿瘤微环境（tumor microenvironment, TME）的调节^[15]^；而富含AT序列的相互作用结构域蛋白1A（AT-rich interaction domain-containing protein 1A, ARID1A）缺失则通过影响染色质重塑及转录调控，改变代谢相关基因表达谱，间接增强糖酵解程序^[16]^。因此，LUAD糖酵解增强并非孤立的代谢事件，而是在多信号通路协同作用下，通过转录放大与代谢级联效应实现的系统性重塑。

### 1.3 TME与表观遗传整合层：乳酸信号与乳酸化调控

在上述细胞内调控框架之外，TME提供了持续的外源性选择压力。缺氧、酸化及营养限制状态可激活HIF-1α依赖的转录网络，上调葡萄糖转运体与糖酵解酶表达，从而增强代谢适应能力^[17]^。乳酸作为糖酵解的主要输出产物，在TME中大量积累并导致局部酸化，可抑制效应T细胞与自然杀伤（natural killer, NK）细胞功能，同时影响血管生成及基质细胞活性，参与塑造免疫抑制性TME^[18]^。结合LUAD细胞在侵袭与转移过程中对糖酵解增强的依赖^[19,20]^，上述现象提示TME压力与糖酵解适应之间存在动态互作（图1）。

**图1:**
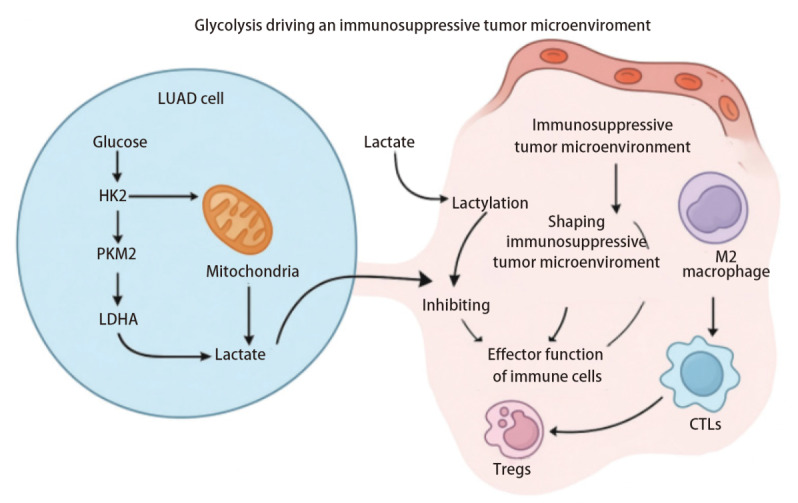
糖酵解增强促进LUAD免疫抑制性微环境形成。增强的糖酵解导致乳酸积累，通过酸化及乳酸化调控抑制CTLs功能，诱导M2型巨噬细胞极化并增强Tregs活性，从而推动免疫抑制与肿瘤免疫逃逸。

近年来的研究进一步揭示，乳酸可作为乳酰基供体介导赖氨酸乳酸化（lysine lactylation, Kla），在代谢与表观遗传调控之间建立直接联系。Zhang等^[21]^首次报道组蛋白Kla可调控基因转录程序，为“代谢——表观遗传耦联”提供分子基础。在肿瘤背景下，组蛋白Kla（如H3K18la）可维持代谢相关基因表达，并参与免疫抑制性转录程序的建立。近年研究^[22]^指出，葡萄糖代谢增强可驱动巨噬细胞组蛋白乳酸化水平升高，从而上调免疫抑制基因表达并促进肿瘤免疫逃逸。虽然相关研究主要来源于特定肿瘤模型，但其所揭示的“代谢——乳酸化——免疫调控”轴为理解LUAD代谢适应与TIME重塑提供了重要机制启示。与此同时，非组蛋白Kla亦被证实可调节代谢酶及信号分子功能，从翻译后修饰层面稳定高糖酵解状态，在NSCLC及其他实体瘤中，Kla被认为参与肿瘤细胞与免疫细胞之间的代谢耦联与功能重塑^[18]^。单细胞与空间组学研究进一步提示，Kla在不同细胞群体中呈现特异性分布，可能参与肿瘤细胞、肿瘤相关巨噬细胞及调节性T细胞之间的免疫代谢互作。然而，在LUAD中特异性Kla底物谱及其因果调控链条仍有待系统阐明。因此，乳酸/Kla构成连接代谢执行层与TME调控层的重要桥梁，使LUAD糖酵解重编程从单纯代谢现象转化为跨层级的调控网络。

综上所述，LUAD中的糖酵解重编程并非单一酶活性增强所致，而是由“关键代谢酶异常激活——信号通路级联放大——TME与表观遗传整合维持”所构成的多层级调控网络。这一分层结构不仅揭示了代谢适应的分子基础，也提示糖酵解依赖性具有可干预性与层级差异性。

从机制层面解析可见，不同层级的调控节点在代谢网络中的功能定位与稳定性并不相同：执行层决定代谢通量输出，调控层影响代谢程序的整体表达框架，而TME整合层则在系统水平维持代谢表型的稳定与反馈强化。因此，针对糖酵解的治疗策略亦不应局限于单一靶点抑制，而需基于其层级结构构建分层干预路径。

在此理论框架下，围绕“执行层直接抑制——调控层级联调节——TME整合干预”的分层策略逐渐成为代谢靶向治疗研究的重要方向。以下将从干预层级角度，系统梳理靶向糖酵解的潜在治疗路径及其临床转化进展。

## 2 靶向糖酵解：LUAD的分层干预与转化路径

### 2.1 执行层干预：关键代谢酶的直接靶向策略

基于糖酵解执行层的异常激活，直接靶向关键限速酶被认为是干预LUAD代谢重编程的基础路径。PDK1、LDHA及HK2等核心节点在维持高糖酵解通量中发挥重要作用，其抑制可在一定程度上恢复氧化代谢比例、降低乳酸生成并削弱代谢输出^[8,23]^。体内外研究^[23]^提示，抑制HK2或LDHA可减弱肿瘤细胞增殖能力并提高代谢应激敏感性。

值得注意的是，部分糖酵解相关酶除催化功能外，还具有转录或信号调节作用。例如，PKM2可发生核转位并参与表皮生长因子受体（epidermal growth factor receptor, EGFR）相关基因转录调控^[24]^；LDHA可通过激活核因子-κB（nuclear factor-kappa B, NF-κB）信号增强细胞存活能力^[25]^；泛素特异性蛋白酶54（ubiquitin-specific protease 54, USP54）通过影响肿瘤抑制因子p53稳定性间接调控糖酵解相关基因表达^[26]^。这些“非代谢功能”拓展了其作为干预靶点的潜在价值。

目前，糖酵解竞争性抑制剂2-脱氧-D-葡萄糖（2-deoxy-D-glucose, 2-DG）已进入早期临床研究，在I期试验^[27,28]^中显示出一定的安全性与可耐受性。然而，由于糖酵解在正常组织中亦具有基础生理功能，其系统性抑制可能带来代谢毒性风险，同时肿瘤细胞可通过代谢可塑性实现补偿。因此，单一代谢酶靶向策略的长期疗效与适用人群仍需进一步明确。

### 2.2 调控层干预：信号通路与转录网络的代谢再编程

鉴于糖酵解受多条上游信号网络协同调控，靶向调控通路或关键转录因子可实现更广谱的“间接代谢抑制”。PI3K/Akt/mTOR信号通路异常激活可促进HK2、LDHA等代谢酶表达并增强葡萄糖摄取；其药物抑制在一定程度上可整体下调糖酵解相关基因表达^[10]^。在缺氧条件下，HIF-1α稳定表达并上调PDK1等关键因子，从而维持低氧环境中的糖酵解优势^[29]^。

此外，癌基因*c-Myc*可直接驱动多种糖酵解相关基因转录，但由于其“难成药”特性，目前更多研究聚焦于其下游依赖网络及可干预节点^[14]^。Notch1-TAZ信号轴可增强糖酵解相关转录并参与免疫逃逸与TME调节^[15]^；RNA结合蛋白SAM68（Src-associated substrate in mitosis of 68 kDa）通过调控PKM可变剪接促进PKM2亚型表达，从而强化Warburg表型^[30]^。与直接酶抑制相比，上游调控通路干预可在转录及信号层面整体降低代谢依赖，但其影响范围更广，也可能伴随更复杂的生物学反馈与耐药机制。

总体而言，调控层干预通过信号——转录——剪接多级网络实现对糖酵解的系统性调节，较单一代谢酶抑制具有更广谱潜力，但其临床应用仍需结合分子背景与代谢依赖特征进行精准分层。

### 2.3 TME整合干预与临床转化前景

在TME与表观遗传整合层面，糖酵解增强所产生的乳酸堆积不仅反映代谢负荷，还参与免疫调节与TME重塑^[18]^。因此，围绕乳酸生成、转运及其关键调控轴的干预策略，被认为有望从更高层级重塑肿瘤代谢稳态与免疫微环境。已有临床前研究^[31]^表明，选择性抑制HK2或LDHA可在一定程度上提高肿瘤细胞对放疗、化疗及靶向治疗的敏感性。然而，糖酵解抑制与其他治疗方式的联合效果仍具有明显的情境依赖性。例如，在部分肿瘤模型中，糖酵解抑制剂与EGFR抑制剂联用后并未表现出稳定一致的协同抗肿瘤效应^[32]^。此外，小分子LDHA抑制剂FX11已在体外研究中显示出抑制有氧糖酵解、降低乳酸生成并抑制肿瘤细胞增殖的潜在作用^[33]^。

从转化角度看，糖酵解靶向策略更具现实意义的模式可能在于与靶向治疗或免疫治疗联合应用，通过降低代谢依赖性增强治疗敏感性。然而，鉴于肿瘤代谢的高度可塑性及TME适应能力，未来研究需结合代谢组学、单细胞测序及代谢影像学进行代谢特征分层，从而提高干预策略的精准性。

## 3 糖酵解相关标志物在LUAD诊断中的应用

### 3.1 组织层标志物：代谢酶及其调控网络

LUAD糖酵解重编程的组织学特征之一是关键代谢酶的持续上调表达。例如HK2在LUAD组织中普遍高表达，其表达水平与肿瘤侵袭性及不良预后密切相关，基于HK2的循环肿瘤细胞（circulating tumor cells, CTCs）检测可识别一类HK2高表达但细胞角蛋白（cytokeratin, CK）低表达或阴性的CTC亚群，该亚群具有较强的代谢活性和侵袭潜能，并与较差生存结局有关^[34]^。LDHA在LUAD组织中亦呈显著上调，其高表达与总生存期缩短存在统计学相关性^[35,36]^。上述结果提示，关键代谢酶不仅反映肿瘤代谢状态，还具有一定的预后分层价值。

除代谢酶本身外，上游调控网络亦参与糖酵解增强。例如，高迁移率族蛋白B1（high mobility group box 1, HMGB1）/SET核蛋白（SET nuclear proto-oncogene, SET）/组蛋白乙酰转移酶1（histone acetyltransferase 1, HAT1）复合物可通过抑制含SAM和SH3结构域蛋白1（SAM and SH3 domain containing 1, SASH1）表达从而增强糖酵解活性^[37]^。AF4/FMR2家族成员4（AF4/FMR2 family member 4, AFF4）通过调控PTEN/PI3K/Akt/mTOR信号通路促进HK2和LDHA表达^[38]^。此外，细胞分裂周期蛋白45（cell division cycle 45, CDC45）在其他肿瘤中被证实与糖酵解增强及不良预后有关^[39]^，提示细胞周期与代谢重编程之间可能存在功能耦联。总体而言，组织层代谢酶及其调控网络的异常表达构成LUAD代谢重编程的重要分子基础，具有潜在的诊断与预后评估价值。

### 3.2 液体活检：循环代谢产物与细胞成分

液体活检为糖酵解相关标志物的动态监测提供了无创手段。代谢组学研究^[40]^显示，LUAD患者血浆乳酸、丙酮酸水平升高，同时伴随色氨酸降低，可形成具有区分能力的代谢特征谱，用于鉴别早期LUAD与良性肺结节。HK2阳性CTCs的检测进一步拓展了代谢标志物的应用范围。研究^[34]^表明，HK2阳性且CK低表达CTC亚群与转移风险升高及不良预后显著相关。由于CTCs可动态反映肿瘤生物学行为，其代谢特征分析在疾病监测及疗效评估方面具有潜在价值。外泌体RNA（extracellular vesicle RNA, EV-RNA）分析亦为早期诊断提供新思路。中国队列研究^[41,42]^发现，血浆外泌体长链RNA（exosomal long RNA, exLR）模型在区分直径<2 cm早期LUAD与良性结节方面具有较高诊断效能，其受试者工作特征曲线下面积（area under the curve, AUC）超过0.90。此外，基于血浆代谢组学特征构建的多变量模型在独立验证队列中AUC可达0.90以上^[43,44]^。

因此，结合HK2阳性CTCs、循环代谢物及EV-RNA特征，有望构建多维联合诊断体系，提高早期筛查与风险分层能力。

### 3.3 影像组学：糖酵解活性的可视化评估

功能影像学为肿瘤糖酵解活性的定量评估提供了重要工具。^18^F-脱氧葡萄糖正电子发射断层显像/计算机断层扫描（^18^F-fluorodeoxyglucose positron emission tomography/computed tomography, ^18^F-FDG PET/CT）通过反映葡萄糖摄取水平评估代谢活性。研究^[45]^发现，天冬氨酰tRNA合成酶2（aspartyl-tRNA synthetase 2, DARS2）表达与PET代谢参数如最大标准摄取值（standardized uptake value maximum, SUVmax）、平均标准摄取值（SUVmean）及总病灶糖酵解量（total lesion glycolysis, TLG）显著相关。此外，糖原沉积水平在LUAD组织中存在空间异质性，其变化可能反映局部代谢适应状态^[46]^。

多模态整合进一步提升诊断准确性。PET参数与CT影像组学特征联合分析可提高孤立性肺结节良恶性鉴别能力^[47]^。动态对比增强磁共振成像（dynamic contrast-enhanced magnetic resonance imaging, DCE-MRI）通过评估血流灌注及TME状态间接反映代谢活性^[48]^。因此，影像学参数与分子标志物联合分析，有助于实现代谢状态的可视化与定量化评估。

### 3.4 多组学整合与临床转化

基于血浆代谢组学构建的多变量模型在LUAD早期筛查中显示出较好的判别能力。独立验证研究^[49,50]^表明，整合多种代谢产物的特征模型在区分早期LUAD与良性肺结节方面具有较高灵敏度和特异性，其AUC可达0.90-0.98。与此同时，HK2阳性CTCs数量与转移风险及不良预后有关^[34]^；程序性死亡配体1（programmed death ligand 1, PD-L1）表达与糖酵解关键酶呈正相关，并与T细胞效应功能呈负相关^[51]^，提示代谢状态可能影响免疫治疗反应。

尽管多组学与液体活检技术具有应用前景，其临床转化仍面临标准化不足、生物学异质性及模型泛化能力受限等问题。未来需在多中心前瞻性队列中验证，并整合代谢组学、转录组学及蛋白组学数据，结合空间组学技术，实现糖酵解活性的精准分型与动态监测^[52]^。多组学融合模型有望提升早期诊断特异性，并为风险分层与治疗决策提供量化依据。

从证据成熟度看，糖酵解相关标志物呈分层发展趋势：^18^F-FDG PET/CT代谢参数及HK2阳性CTCs已具一定临床基础；血浆代谢特征模型与exLR模型处于临床验证阶段；Kla修饰位点及单细胞代谢评分体系仍处于机制探索阶段。分层整合不同层级指标，有助于构建覆盖组织、循环及影像维度的多模态代谢评估体系，推动LUAD向“分子——代谢整合”精准分型转变。

## 4 总结与展望

综合前述机制研究与多组学证据，糖酵解重编程不仅是LUAD代谢适应的核心表型，更是连接肿瘤进展、免疫逃逸及治疗反应差异的重要枢纽。其由代谢执行、信号调控及TME与表观遗传整合构成的多层级网络，使肿瘤在缺氧及治疗压力下维持持续生存优势。

相较于传统影像学及单一分子标志物，糖酵解相关指标可更早反映功能状态变化，并具备动态监测潜力。将代谢特征纳入风险分层模型有助于提升早期诊断及疗效预测能力，使糖酵解由单纯代谢现象转变为具有临床价值的分型依据和潜在治疗靶点。

在转化层面，靶向糖酵解关键酶及其上游信号通路在临床前研究^[53,54]^中已显示抑瘤作用，单细胞测序研究揭示LUAD TME中存在显著细胞异质性，而基于代谢影像的研究^[55]^进一步表明肿瘤代谢过程具有动态及空间差异特征，提示未来可基于代谢分型实施个体化治疗。然而，其临床应用仍面临检测标准化不足、多组学整合有限等挑战，尤其是乳酸及组蛋白乳酸化相关指标尚缺乏统一检测体系。此外，尽管^18^F-FDG PET/CT已广泛应用于代谢评估，但对乳酸代谢通量及空间异质性的反映仍有限。新兴代谢组学技术及人工智能辅助影像分析有望提升代谢状态评估与风险预测能力^[56,57]^。

总体而言，LUAD糖酵解研究正处于由机制探索向临床转化推进的关键阶段。未来通过完善检测体系、深化多组学整合并融合智能影像技术，有望构建以代谢重编程为核心的精准诊疗模式，实现风险分层与个体化干预。
